# How are the village health volunteers deliver malaria testing and treatment services and what are the challenges they are facing? A mixed methods study in Myanmar

**DOI:** 10.1186/s41182-018-0110-0

**Published:** 2018-08-02

**Authors:** Nay Yi Yi Linn, Jaya Prasad Tripathy, Thae Maung Maung, Khine Khine Saw, Lei Yee Win Maw, Badri Thapa, Zaw Lin, Aung Thi

**Affiliations:** 1 Vector Borne Disease Control Program, Ministry of Health and Sports, Nay Pyi Taw, Myanmar; 2 International Union Against Tuberculosis and Lung Disease, The Union South East Asia Office, New Delhi, India; 3 International Union Against Tuberculosis and Lung Disease, Paris, France; 4 Department of Medical Research, Ministry of Health and Sports, Yangon, Myanmar; 5 Independent Public Health Consultant, Yangon, Myanmar; 6 Malaria Unit, World Health Organization Country Office, Yangon, Myanmar

**Keywords:** Community health workers, Midwife, Rapid diagnostic test, Undifferentiated fever

## Abstract

**Background:**

Village health volunteers (VHVs) play a key role in delivering community-based malaria care especially in the hard-to-reach areas in Myanmar. It is necessary to assess their performance and understand the challenges encountered by them for effective community management of malaria. This mixed methods study was conducted to (i) understand the cascade of malaria services (testing, diagnosis, referral, and treatment of malaria) provided by the VHVs under the National Malaria Control Programme (NMCP) in Myanmar in 2016 and compare with other health care providers and (ii) explore the challenges in the delivery of malaria services by VHVs.

**Methods:**

A sequential mixed methods study was designed with a quantitative followed by a descriptive qualitative component. The quantitative study was a cohort design involving analysis of secondary data available from NMCP database whereas the qualitative part involved 16 focus group discussions (eight each with community and VHVs) and 14 key informant interviews with program stakeholders in four selected townships.

**Results:**

Among 444,268 cases of undifferentiated fever identified by VHVs in 2016, 444,190 were tested using a rapid diagnostic test. Among those tested, 20,375 (4.6%) cases of malaria were diagnosed, of whom 16,910 (83.0%) received appropriate treatment, with 7323 (35.9%) receiving treatment within 24 h. Of all malaria cases, 296 (1.5%) were complicated, of whom 79 (26.7%) were referred to the higher facility. More than two thirds of all cases were falciparum malaria (13,970, 68.6%) followed by vivax (5619, 27.6%). Primaquine was given to 83.6% of all cases. VHVs managed 34.0% of all undifferentiated fever cases, 35.9% of all malaria cases, and identified 38.0% of all *Plasmodium falciparum* cases reported under NMCP. The key barriers identified are work-related (challenges in reporting, referral, management of malaria especially primaquine therapy, and lack of community support) and logistics related (challenges in transportation, financial constraints, time and shortage of drugs, and test kits). On the other hand, they also enjoy good community support and acceptance in most areas.

**Conclusion:**

VHVs play an important role in malaria care in Myanmar, especially in the hard-to-reach areas. More programmatic support is needed in terms of logistics, transportation allowance, and supervision to improve their performance.

## Background

Malaria is a serious public health threat because of its severity and often fatal outcome. Over half of the population is known to be at risk of malaria globally. The World Health Organization (WHO) reported 216 million cases and an estimated 445,000 deaths due to malaria in 2016 globally. Asia and Pacific regions cover 22 countries with 2.1 billion people, of which 80% are at risk of getting malaria [1].

WHO Global Technical Strategy for Malaria 2016–2030 has set a target of 90% reduction of malaria cases by 2030 compared to 2015 to combat malaria [2]. Sustainable Development Goal 3.3 also envisages ending the malaria epidemic by 2030 [3].

Myanmar has a high burden of malaria with more than two thirds of its population at risk of malaria [4]. Myanmar, located in the tropical zone, provides a favorable wet and moist climate for breeding of *Anopheles* mosquito. Nearly three fourths of the cases of malaria are caused by *Plasmodium falciparum* [5, 6]. It is also one of the countries in Greater Mekong Subregion (GMS) with documented evidence of artemisinin resistance [7]. In line with the global strategies, the National Malaria Control Programme (NMCP) in Myanmar is also committed to eliminate malaria by 2030 by ensuring equitable and universal access to effective malaria preventive and curative services [4].

Community health workers (CHWs) have an important role to play in achieving the global malaria targets. A systematic review by Paintain et al. has supported the CHWs to implement effective community case management (CCM) for malaria [8]. A dedicated workforce like the placement of village health volunteers (VHVs) was proven successful in improving the access to and utilization of malaria testing and treatment services in a cluster randomized trial in Myanmar [9]. In line with this, VHVs are important actors in malaria control and elimination in Myanmar especially in the hard-to-reach areas with poor formal public health infrastructure. Myanmar introduced VHVs in 2007 at the community level to provide universal access to malaria prevention and care services among rural and hard to reach populations [10]. They constitute the grassroot-level workers in the public health system. In Myanmar, community-based malaria diagnosis and treatment services are primarily delivered by a network of around 15,000 VHVs (~ 9000 under the NMCP and remaining under various local and international nongovernmental organizations). VHVs are involved in the early diagnosis of malaria (using rapid diagnostic test, RDT) and prompt treatment (using artemisinin combination therapy (ACT)/chloroquine and primaquine) which is the cornerstone of malaria control. They are also involved in promoting awareness about the prevention and management of malaria, distribution of insecticidal-treated bed nets, and referral of complicated cases [4].

As Myanmar heavily relies on VHVs in delivering community-based malaria diagnostic and treatment services especially in the hard-to-reach areas, it is necessary to assess their performance and understand the challenges encountered by them. Currently, there is little information on how the VHVs are performing in terms of their contribution to CCM of malaria [11].

Although few studies in Myanmar have qualitatively explored the challenges encountered by the volunteers, those that exist are either limited to the use of RDTs or restricted to specific geographical areas [12, 13]. As Myanmar is committed to eliminate malaria in six states/regions by 2020 (hereafter referred to as malaria elimination areas) and in the remaining states/regions by 2030 (hereafter referred to as malaria transmission reduction areas), it would also be useful to understand the challenges faced by the VHVs in both these settings [11].

Thus, the present study was conducted with the following specific objectives: (i) to understand the cascade of care (testing, diagnosis, referral, and treatment of malaria) among cases of undifferentiated fever identified by the VHVs under the NMCP in Myanmar in 2016 and to compare with other malaria care providers/sources of malaria cases and (ii) to explore the challenges in the delivery of malaria diagnostic and treatment services by VHVs and understand the community acceptance of VHVs.

## Methods

### Study design

The study design contains a sequential QUAN-QUAL mixed methods study with a quantitative (involving analysis of secondary data available from the National Malaria Control Programme database) followed by a descriptive qualitative component.

### Study setting

#### General setting

The Republic of the Union of Myanmar, located in Southeast Asia, is bounded by Bangladesh, India, China, Laos, and Thailand. The country is divided administratively into Nay Pyi Taw Council Territory and 14 regions/states. It consists of 74 districts and 330 townships with a population of around 51.5 million [4, 14]. The Ministry of Health and Sports, Myanmar, delivers health services through its basic health services under the Department of Public Health and the Department of Medical Services.

#### Specific setting

Malaria is endemic in 291 out of 330 townships in Myanmar with nearly 44 million at risk of malaria. The NMCP carries out malaria control activities in line with the global and national malaria control strategies in collaboration with national and international partners [4]. The main malaria-related interventions include community awareness, insecticide-treated bed net distribution, indoor residual spraying, early diagnosis of malaria using RDT and microscopy among persons with undifferentiated fever, and ACT-based treatment, all of which are provided free-of-charge [11]. The National Malaria Elimination Plan in Myanmar (2016–2030) envisages *Plasmodium falciparum* elimination throughout the country by 2025 and a malaria-free Myanmar by 2030 [11].

**Fig. 1:**
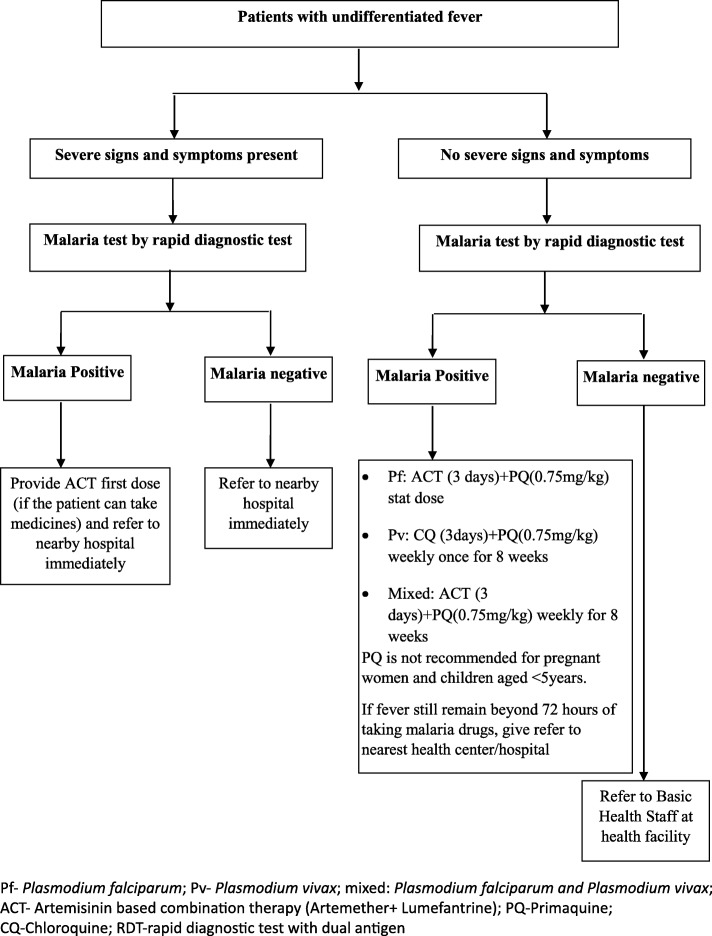
Malaria testing, diagnosis, and treatment services provided by village health volunteer in Myanmar, 2017

Integrated malaria care services are delivered both at the health facility and community level by basic health staff (BHS) and VHVs, respectively. VHVs are recruited by the NMCP at a village level in hard-to-reach and conflict-stricken areas. They are trained by the NMCP and other implementing partners (IPs). The training lasts for 5 days for a new recruit and 2 days for a refresher course. They are supervised by the BHS in the respective areas. They provide malaria diagnosis using RDT dual antigen (*Plasmodium falciparum* and *Plasmodium vivax*) and treatment according to the national malaria treatment guidelines. However, they do not follow up with the patients to ensure treatment compliance. The VHVs obtain drugs and diagnostic kits from the sub-rural health centers. The algorithm of testing and management of malaria by VHV is given in Fig. 1 [11, 15]. VHV should refer all pregnant women, children less than 1 year with malaria, and complicated malaria cases to BHS of higher health facility for further treatment. NMCP provides 60,000 MMK (US$50) as a quarterly incentive for the services delivered by VHVs. BHS are the key health care providers in rural areas and comprise of public health supervisors, midwives, lady health visitor and health assistants at rural health centers, and/or sub-centers. The BHS is responsible for maternal and child health, school health, nutritional promotion, immunization, community health education, environmental sanitation, disease surveillance and control, treatments of common illnesses, referral services, birth and death registration, and training of village health volunteers [10, 16]. VHVs submit the monthly case report to the BHS stationed at the respective sub-rural center with details of the persons with undifferentiated fever tested, diagnosed, and treated for malaria, which is then transmitted to the township health department. Aggregate data from the township are entered in an Excel-based format by data focal person and reported to the respective states and regions. Data assistants in the states and regions collate data from different townships and then transmit to the central NMCP office at Nay Pyi Taw [4, 15].

### Study population

For the quantitative study, the study population included a cohort of all patients with undifferentiated fever tested, diagnosed, and treated for malaria under the NMCP in Myanmar in 2016. For assessing the performance of VHVs, only VHVs under the NMCP were studied.

For the qualitative study, VHVs, BHS, NMCP staff, and community members in four selected townships of four states/regions were selected in 2017.

### Data variables, sources of data, and data collection

#### Quantitative component

Data were extracted from the National Malaria electronic (Excel-based) case register in 2016. The data variables included age, sex, state/region, source of case, type of test (RDT/microscopy), test result, type of malaria species (*Plasmodium vivax*/*P*. *falciparum*/mixed/others—*P*. *ovale* and *P*. *malariae*), severity of malaria (complicated/uncomplicated), timing of treatment (within 24 h or not), and type of treatment offered, whether referred (yes/no).

Operational definitions of key terms and other derived variables are described in Table 1.

**Table 1:** Operational definitions of key terms and derived variables

Undifferentiated fever	The occurrence of fever in a person in the last 2 weeks
Malaria case	Occurrence of malaria infection in whose blood the presence of malaria parasites (*Plasmodium*) has been confirmed by a diagnostic test (rapid diagnostic test/microscopy).
Basic health staff (BHS)	They are salaried employees working in sub-center/rural health center/township health departments (urban and rural areas) and are responsible for malaria prevention, control, and treatment. They are also involved in the control and prevention of other diseases such as tuberculosis, maternal and child health, other vector-borne diseases and immunization.
Village health volunteer (VHV)	They are incentive-based community volunteers and deployed in hard-to-reach areas to provide screening, diagnosis, and treatment of malaria in the communities. They are involved in providing health education, malaria diagnosis and treatment to the communities, and referral of severe cases to higher health facilities. VHVs are employed by the NMCP and other international nongovernmental organizations. NMCP provides 60,000 MMK (US$50) as quarterly incentive for the services delivered.
Malaria species	Malaria species refers to the type of malaria parasite: *Plasmodium falciparum*, *Plasmodium vivax*, others (*Plasmodium malariae* and *P*. *ovale*), and mixed infections
Severe malaria (complicated malaria)	Patients with complicated malaria, also known as severe malaria, are categorized when the patients develop the following signs and symptoms: cannot walk, sit, or stand without help; vomit and cannot take a medicine; high-grade fever; severe anemia or pallor; yellow coloration of the sclera or jaundice; behavioral changes; confusion; anger; drowsiness; slurred speech; coma; breathlessness and tightness of the chest; cold and clammy extremities and shock; reduced urine output or anuria; hematuria or other bleeding manifestations; and fever continuation for > 72 h of medication Patients with uncomplicated malaria will not develop any symptoms and signs of severe malaria
Appropriate treatment	Treatment including both schizonticidal and gametocidal drugs according to the national malaria treatment guidelines
Other malaria care providers/source of malaria cases	They included basic health staff, Mobile Medical Unit, malaria clinic, screening point, active case detection, intensified case detection

Other malaria care providers/sources of malaria case include BHS, active/intensified case detection, Mobile Medical Unit, surveys, malaria clinic, and other screening points.

#### Qualitative component

The challenges in the delivery of malaria diagnostic and treatment services by the VHVs were explored through focus group discussions (FGDs) with VHVs and key informant interviews (KIIs) with BHS and vector-borne disease control (VBDC) staff. FGDs were also conducted with the community members to understand their acceptance on the role of VHVs in malaria control in the village.

The principal investigator (PI) along with a team of trained qualitative researchers conducted the KIIs and FGDs during daytime and in place convenient to the participants, preferably nearby township health department or the community after obtaining written informed consent. Participants were informed of the purpose of the study before the data collection. Only participant(s) and the researcher were present during the KII/FGD sessions. FGDs lasted an average of 40 min while KIIs lasted an average of 30 min. The PI did not have a prior relationship with any of the participants and was not involved in the provision of the medical care of the community members. A program volunteer introduced the PI to the participants but was not present during the interviews. Pilot-tested KII and FGD guides with broad open-ended questions were used to conduct the interviews and group discussions. The KIIs/FGDs were audio-recorded and supported by a Burmese-speaking notetaker. Verbatim notes and memos were written by the PI during data collection. These were carried out between January to February 2018.

Fourteen KIIs were conducted with health care providers in four selected townships, namely Singu, Bilin, Hlaingbwe, and Pathein, one each from malaria elimination (i.e., Mandalay and Mon) and transmission reduction states/regions (i.e., Ayeyarwaddy and Kayin). They were conveniently selected based on their availability and willingness to participate.

Two FGDs were conducted with the VHVs under the NMCP in each of the four townships (a total of eight FGDs). Similarly, two FGDs were conducted with the community members in each of the four townships (a total of eight FGDs). Around six to ten participants were present in each FGD. Convenience sampling was used to recruit these participants.

### Data analysis and statistics

#### Quantitative

Quantitative data in Excel format was extracted from the NMCP database, cleaned, and imported into EpiData analysis version 2.2.2.182 for analysis (EpiData Association, Odense, Denmark). The number and proportions were used to describe the malaria diagnostic and treatment care cascade. Proportions were also calculated to describe the type of malaria species, severity, and treatment of malaria.

#### Qualitative

Audio-recorded tapes from FGDs and KIIs were manually transcribed in the local language (Burmese) as soon as the data collection was over. The transcripts were read several times by the PI along with another co-investigator (LYWM) and coded with the aid of Atlas.ti software (6.2). Codes were discussed between the co-investigators to clarify any differences of opinion. Discrepancies were resolved through discussion and referral back to the original audio files wherever necessary. The codes were then organized to generate common themes [17, 18].

### Ethical considerations

Ethical approval for the study was obtained from the Ethical Review Committee (ERC) of the Department of Medical Research, Myanmar (Ethics/DMR/2018/030) and from the Union Ethics Advisory Group of the International Union of Tuberculosis and Lung Disease (The Union), Paris, France (75/17). Administrative permission for the study was sought from the National Malaria Control Programme (NMCP), Myanmar. Written informed consent was obtained from the participants for the participation in interviews and focus group discussions, and a separate written consent was also obtained for audio-recording the proceedings.

## Results

**Fig. 2:**
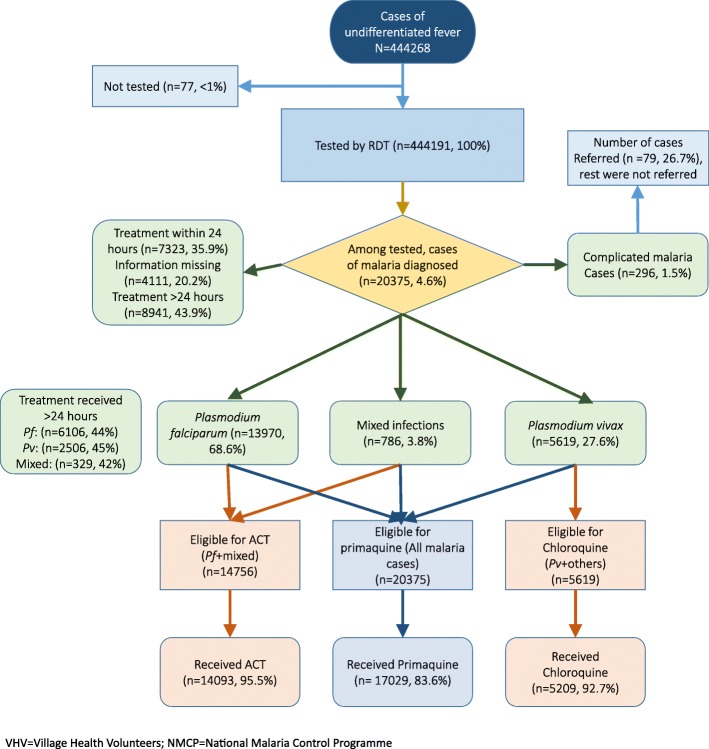
Flowchart showing the cascade of malaria care (testing, diagnosis, and treatment) among patients with undifferentiated fever identified by VHVs under NMCP in Myanmar, 2016

Among 444,268 cases of undifferentiated fever identified in 2016 by the VHVs under NMCP, almost all (*n* = 444,190) of them were tested using RDT. Among those tested, 20,375 (4.6%) cases of malaria were diagnosed, of whom 296 (1.5%) were complicated malaria cases and 7323 (35.9%) were initiated on treatment < 24 h. There was no information on 4111 (20.2%) cases of malaria about the timing of treatment initiation. Of the complicated malaria cases, 79 (26.7%) were referred to the higher facility. More than two thirds of all cases were falciparum malaria (13,970, 68.6%) followed by vivax (5619, 27.6%) and mixed infections (786, 3.8%). Of those eligible for ACT (*n* = 14,756), choloroquine (*n* = 6619), and primaquine (*n* = 20,375), a total of 14,093 (95.5%), 5209 (92.7%), and 17,029 (83.6%) received the treatment, respectively (Fig. 2).

**Fig. 3:**
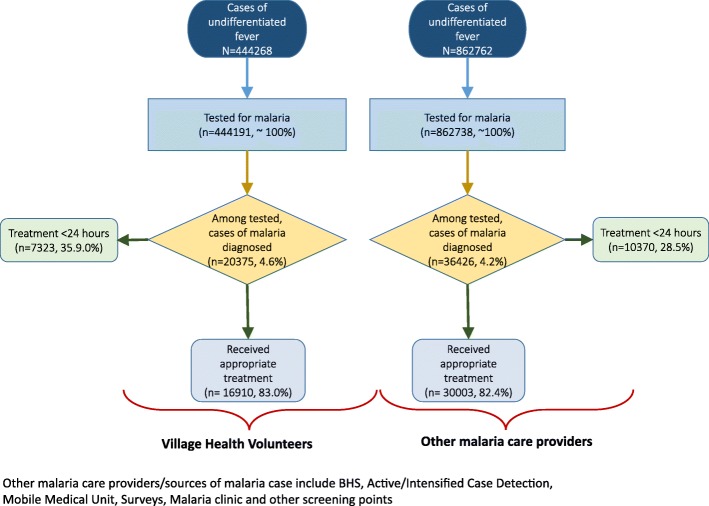
Flowchart comparing the cascade of care (testing, diagnosis, and treatment) by village health volunteers and other malaria care providers under NMCP in Myanmar, 2016

Figure 3 shows that the performance of VHVs is comparable to that of other malaria care providers with respect to malaria diagnostic and treatment services. The initiation of anti-malarial treatment within 24 h is higher among cases diagnosed and treated by VHVs (35.9%) compared to other malaria care providers (28.5%) (*p* < 0.001).

**Fig. 4:**
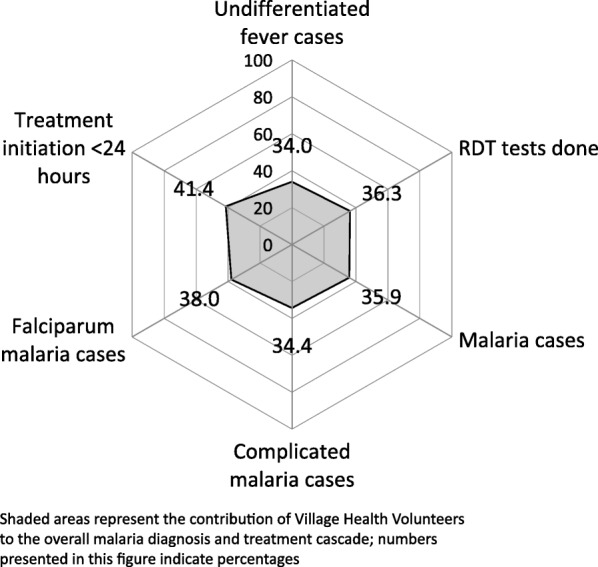
Contribution of village health volunteers in malaria diagnosis and treatment activities under the National Malaria Control Programme in Myanmar, 2016

Within the overall malaria diagnosis and treatment cascade in 2016 in Myanmar, VHVs were responsible for managing 34.0% of all undifferentiated fever cases and 35.9% of all malaria cases identified under the NMCP in 2016. They also identified 38.0 and 34.4% of all *Plasmodium falciparum* and complicated malaria cases, respectively (Fig. 4).

Table 2 shows the demographic characteristics of undifferentiated cases of fever and malaria cases identified by the VHVs. Nearly 30% of the malaria cases are children (< 15 years of age), and two thirds are males. A majority of malaria cases (57.6%) resided in Rakhine State.

**Table 2:** Demographic characteristics of undifferentiated fever and malaria cases identified by village health volunteers in Myanmar, 2016

Characteristics	Undifferentiated fever cases		Malaria cases	
	*N*	%	*N*	%
Total	444,268	100	20,375	100
Age group				
< 1 year	4219	0.9	124	0.6
1–4 years	28,248	6.4	1729	8.5
5–9 years	45,527	10.2	2270	11.1
10–14 years	44,134	9.9	2136	10.5
≥ 15 years	322,084	72.5	14,116	69.3
Missing	56	< 1	0	0.0
Sex				
Male	237,129	53.4	13,563	66.6
Female	207,084	46.6	6812	33.4
Missing	55	< 1	0	0.0
State/region				
Ayeyarwaddy	18,899	4.3	1796	8.8
BagoE	35,600	8.0	115	0.6
BagoW	12,453	2.8	22	0.1
Magway	16,490	3.7	65	0.3
Mandalay	7576	1.7	356	1.7
Nay Pyi Taw	2112	0.5	124	0.6
Sagaing	20,594	4.6	1511	7.4
Tanintharyi	19,461	4.4	443	2.4
Yangon	25,536	5.7	5	< 1
Chin	13,808	3.1	1590	7.8
Kachin	9823	2.2	384	1.9
Kayah	16,163	3.6	120	0.6
Kayin	16,480	3.7	225	1.1
Mon	11,641	2.6	94	0.5
Rakhine	92,403	20.8	11,736	57.6
ShanE	9941	2.2	530	2.6
ShanN	32,656	7.4	239	1.2
ShanS	82,632	18.6	1020	5.0

**Fig. 5:**
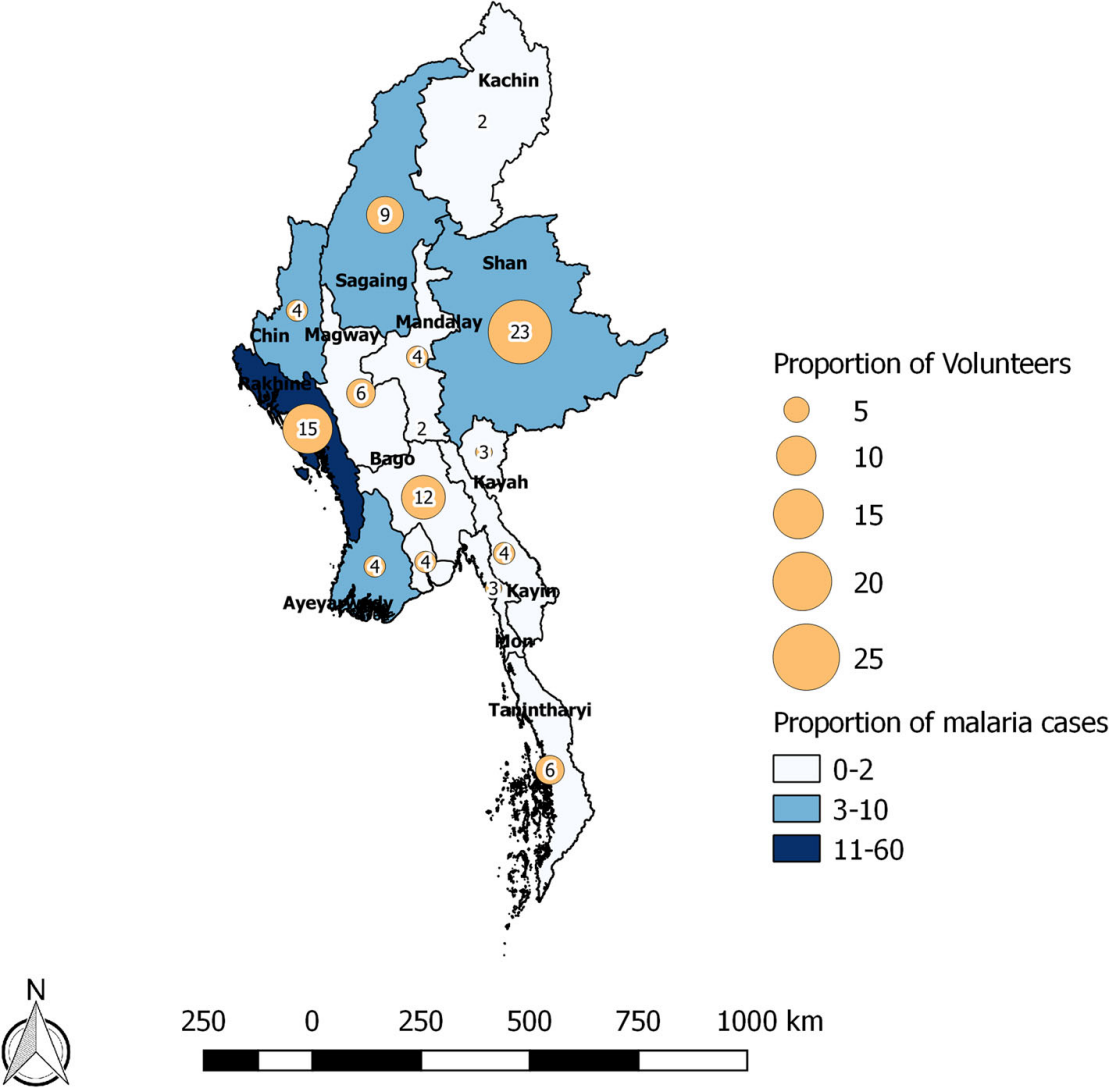
Map of Myanmar showing the distribution of village health volunteers and malaria cases identified by them in different states and regions, 2016

Figure 5 shows the distribution of VHVs and the proportion of malaria cases in different states/regions. States like Shan and Bago contribute to ~ 10% of all malaria cases but have nearly 35% of all VHVs. On the other hand, Rakhine, which contributes to ~ 58% of all malaria cases, have 15% of VHVs posted.

### Qualitative results

Table 3 shows the socio-demographic characteristics of the FGD and KII participants. A total of eight FGDs were conducted with the community members involving 66 participants. They were mostly farmers (*n* = 32) and laborers (*n* = 19). A total of eight FGDs were conducted with the volunteers involving 65 participants. Fourteen KIIs were carried out with key VBDC staff such as malaria supervisor (*n* = 2), malaria inspector (*n* = 1), malaria assistant (*n* = 1), Regional Malaria Officer (assistant director) (*n* = 1), and other health care providers such as BHS (*n* = 5) and health assistant (*n* = 4).

**Table 3:** Socio-demographic characteristics of the participants of the focus group discussions and key informant interviews conducted in four selected townships in Myanmar, 2017

Characteristics	FGDs with volunteers		FGDs with community^a^		KII participants^b^	
	*N*	%	*N*	%	*N*	%
Total	65	100	66	100	14	100
Gender						
Male	25	38	18	27	6	43
Female	40	62	48	73	8	57
Age group						
15–24 years	10	15	9	14	0	0
25–44 years	43	66	31	47	8	57
45–64 years	11	17	22	33	6	43
65 years and above	1	2	4	6	0	0
Education						
Up to primary	5	8	53	80	0	0
Up to high school	52	80	13	20	2	14
Graduate	8	12	0	0	8	57
Missing	0	0	0	0	4	29
Years of service						
0–5 years	37	57			3	21
6–10 years	24	37			3	21
More than 10 years	3	5			6	43
Missing	1	2			2	14

^a^Community members were fishermen (*n* = 3), farmer (n = 32), laborer (*n* = 19), trader (*n* = 3), and dependent members (*n* = 9)

^b^KII participants were midwives (*n* = 5), malaria inspector (*n* = 1), health assistant (*n* = 4), malaria supervisor (*n* = 2), malaria assistant (*n* = 1), and assistant director (*n* = 1); FGD = focus group discussion; KII = key informant interview

**Fig. 6:**
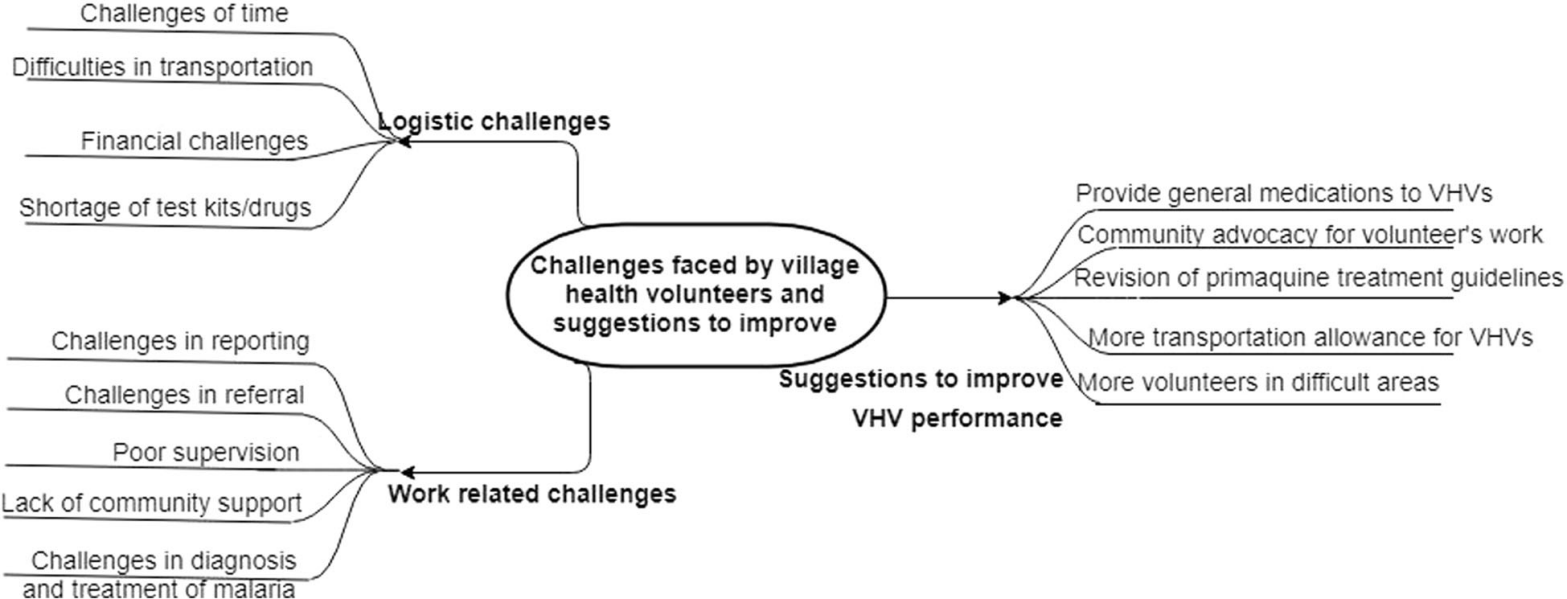
Thematic diagram showing the themes and sub-themes around challenges faced by village health volunteers, community acceptance for volunteers and suggestions to improve their performance in Myanmar, 2017

Four overarching themes emerged from the KIIs and FGDs relating to (a) work-related challenges, (b) logistics challenges, (c) community perception and acceptance for VHVs, and (d) suggestions to improve the performance of VHVs (Fig. 6).

### Theme 1: Work-related challenges

#### Challenges in reporting

The VHVs have to report to the BHS monthly. Although many of them were able to report timely, few could not because of the difficulty in reaching the reporting facility due to the hard-to-reach locations and lack of transportation facilities. For case notification within 24 h, some of the volunteers who lived in difficult terrains with poor phone connectivity were not able to report within 24 h. A midwife explained:

“They submit the reports to the midwives. Some volunteers could not report case within 24 hours because of poor phone connectivity and transportation issues. If they had phone service, they would have reported within 24 hours” (KII: 31 years, F, midwife).

A 33-year-old male health assistant substantiated these challenges in reporting by saying:

“Some volunteers are from hard to reach areas; they could submit reports 3 monthly. Volunteers from other areas usually report on a monthly basis” (KII: 33 years, M, health assistant).

Another volunteer reported difficulty in filling the carbonless register, as she said:

“We have faced challenges in filling carbonless registers, and asked the midwives for help to fill them. We usually record the data first in the book and then transfer the data to the register” (FGD: 34 years, F, VHV).

#### Challenges in referral

Few volunteers said that patients after referral did not go to the referred health center because of their misconceptions, poor transportation, and financial constraints. Some of the volunteers who accompanied complicated cases of malaria to the referred facility had to arrange or sometimes bear the costs of transportation, as this 42-year-old volunteer said:

“We have to go along with the patient for referral and thus bear the extra cost for fuel; those who do not have motorbike spend more money on transportation” (FGD: 42 years, M, VHV).

#### Challenges in supervision

Poor supervision was also cited as one of the challenges by this volunteer:

“We meet the supervisors once a year as they are busy in their own work and do not have time to supervise us” (FGD: 36 years, M, VHV).

#### Challenges in community support

Some of the volunteers faced difficulty in dealing with the community as they reported poor support from the village leaders who showed the least interest in health activities. Few volunteers complained that they were not given due respect by the community members because they belonged to the same village in accordance with the saying “Familiarity breeds contempt.”

“The community did not show respect if we were alone. When the BHS would introduce us to the community, they start giving some respect. They would not listen to us as we are from the same locality, like the saying goes The grass on the other side of the river appears greener” (FGD: 33 years, F, VHV).

Language barrier in some states like Kayin was highlighted by this midwife:

“In Kayin state, volunteers were scared to go and speak with people as they don’t understand Burmese language. Language was the main barrier and they only trusted the volunteers who could speak Kayin language” (KII: 31 years, F, midwife).

#### Challenges in diagnosis and treatment of malaria

Although most of the volunteers were familiar with the use and interpretation of RDTs, few volunteers were not confident in using them, especially among young children.

“We face difficulties in testing RDT for young children” (FGD: 27 years, F, VHV).

A 41-year-old midwife raised the following concern regarding the guidelines for primaquine therapy for non-falciparum infection.

“Our non-pf primaquine therapy guidelines are different from those meant for the volunteers. We give primaquine for 14 days daily, but for the volunteers, they are instructed to give primaquine once per week for 8 weeks” (KII: 41 years, F, midwife).

Ensuring the completion of an 8-week primaquine therapy was quite challenging, as this volunteer explained:

“We have difficulties in administering primaquine for 8 weeks and ensuring treatment compliance. The patient might not stay in the same place. They also tend to forget to take the drugs weekly for 8 weeks” (FGD: 67 years, M, VHV).

### Theme 2: Logistic challenges

#### Transportation challenges

Because most of the volunteers belong to the hard-to-reach areas, they face transportation difficulties especially during the rainy season. Security is also a major concern for them especially in these inaccessible areas. The supervisors also stated that volunteer drop out was common in these areas.

“We face difficulty in transportation during the rainy season as we have to travel for a day and cross the river. We have to come one or two days prior to the meeting” (FGD: 46 years, F, VHV).

#### Financial challenges

The volunteers had to bear the cost of travel for reporting, accompanying the patients for referral and reaching the migrant sites. Few volunteers have also left the job because of this.

“We have difficulties in transportation. We would like to request reimbursement of transportation charges for the volunteers” (KII: 31 years, F, midwife).

“The volunteers are not getting their incentives monthly, rather quarterly, so they look for other jobs for their living” (KII: 47 years, M, malaria supervisor).

#### Challenges of time

Although most of them had no difficulties in contributing time for their volunteer work, few, who had other personal/family issues, faced challenges with regard to managing their time for this job.

“Some volunteers are engaged in another job for their living and thus, could not give much time for volunteer work” (KII: 50 years, F, midwife).

#### Shortage of drugs and RDT test kits

Although most volunteers have reported the adequate availability of RDTs, few of them have also mentioned stock out of RDTs and ACT especially in the hard-to-reach areas and during the rainy season.

“Sometimes we have shortage of ACT and RDT. Once during the rainy season, there were lots of fever cases and we could not perform RDT testing for about 1 or 2 months” (FGD: 36 years, M, VHV).

### Theme 3: Community perception and acceptance for VHVs

#### Community perception and practice towards malaria treatment

The volunteers and BHS responded that people generally resort to self-medication or approach quacks first if they get a fever and test for malaria only if the fever does not subside. Few of them even said that things have changed now. Some communities approach the VHVs and get themselves tested for malaria immediately upon getting a fever.

“Before they seek treatment from us, they take self-medications or seek treatment from quacks. They come to us only when they do not get relief” (FGD: 63 years, M, VHV).

“Few communities seek treatment from the volunteers, some go to a hospital/clinic and some approach a quack. The number of quacks are more than the number of volunteers” (KII: 33 years, M, health assistant).

#### Community acceptance for the volunteers

Most of the volunteers are accepted by the community because they are native people living in the same village, could communicate with the local language and they are involved in other health matters, too. The community accepted the malaria testing, treatment, and health education session done by the volunteers as the diagnosis and treatment of malaria were free of charge. In Hlaingbwe township, there were few villages that did not accept the volunteers at all in their village because the villages are served by their ethnic organization.

“Most of the villagers go to the volunteer when they have fever. Service is free of charge so the villagers have less to spend.”

“He also helps in taking the patient to the hospital. He is very helpful, supportive and the whole village likes him. We can discuss him about health matters (whether good or bad things)” (FGD: 37 years, M, VHV).

#### Community support to the volunteers

Most of the volunteers get good support from the community and village leaders. The village leaders would help them in mobilizing the community for health educations and immunization and sometimes arrange their transportation as well. The community also recognizes their role in improving the health of the villagers. A volunteer in an FGD said:

“The village leaders help us, they recruit villagers, one from each household for health education sessions and motivate the community to attend the sessions” (FGD: 33 years, F, VHV).

#### Community preference towards volunteer selection

Some communities preferred volunteers who were more educated and could allocate time for volunteer work. However, they had no age and gender preference for the volunteers.

“There are about 10 youths in the village who are graduates, but without a job. If they are selected as volunteers, it would be better for us” (FGD: 63 years, M, farmer).

### Theme 4: Suggestions to improve performance of VHVs

Some health staff suggested supporting the volunteers with some medicines like paracetamol, oral rehydration salt, and multivitamins for general ailments to build the community trust upon them.

“It would be better if we can provide paracetamol, ORS and multivitamins to the volunteers” (40 years, M, malaria inspector).

Village leaders and community should be advocated about the volunteers’ work by arranging a village meeting or during the health activities done by the BHS to ensure community support. More financial allowance should be given for the volunteers to support their transportation costs and more volunteers should be assigned to the hard-to-reach areas.

## Discussion

This is the first mixed methods study assessing the performance of VHVs in Myanmar and the challenges they encounter in delivering malaria care services. There were some interesting findings.

Firstly, it was encouraging to note that more than 90% of malaria patients have received treatment similar to the figures reported in a systematic review [8]. However, only one out of three patients with malaria is initiated on treatment within 24 h of diagnosis by the VHVs which is much less than the WHO target of at least 60% [19]. This is worrisome as delayed treatment initiation might lead to complications and death. This could be due to the user- or provider-related factors. Poor treatment-seeking behavior on the part of the user has been reported earlier in similar settings [20, 21]. The preference for self-medication and informal health care providers has also been reported previously [21, 22]. This is also substantiated by the qualitative findings in this study which state that people generally approach VHVs when they do not get relief from self-medication and other informal care providers, thereby losing the crucial initial 24 h in the process.

On the provider side (i.e., VHVs), backed by the qualitative findings, we speculate that the logistics challenges like shortage of test kits, challenges of transportation, and time are some of the reasons for delayed treatment initiation. Due to financial constraints, some of the volunteers also do other jobs outside their village which might explain this delay. There was no information on the timing of treatment initiation in one fifth of the cases. This could be due to the challenges in filling the carbonless registers (malaria case register) and poor supervision as reported by the VHVs. This can be corrected by checking during routine supervision and providing on-site training.

Secondly, the primaquine was used in eight out of ten patients. However, we should be aiming for 100% radical cure in order to achieve malaria elimination. Adherence to this drug has commonly been poor as reported in previous studies [23, 24]. This is probably because the program’s focus has generally been on acute anti-malarial treatment to eliminate the initial threat to the patient (i.e., from blood stage parasites) rather than preventing future recurrences [23]. Another probable reason for this finding came out of the qualitative interviews with health care providers. The BHS said that the guidelines for primaquine therapy in case of non-falciparum malaria are different for VHVs and BHS which adds to the complexity of the management. VHVs have to give primaquine weekly for 8 weeks, whereas for other health staff, it is once daily for 14 days due to the concerns around G-6PD deficiency and associated complications. They have also felt that it is difficult to ensure the completion of an 8-week primaquine therapy. Future trainings and supervisory visits should focus more on radical cure management and its importance in achieving malaria elimination.

Thirdly, complicated cases of malaria are at high risk of dying which requires referral to a higher facility. However, the referral of complicated cases by VHVs has been poor which has also been documented in previous studies from the African region [25]. This is an important aspect of CCM of malaria in order to achieve and sustain zero malaria deaths which needs to be re-emphasized during program meetings and trainings at various levels. The volunteers have identified two reasons for this during the in-depth exploration: (i) patients after referral do not go to the referred health center due to their beliefs, poor transportation, and financial constraints and (ii) accompanying them to the health center would entail substantial transportation costs to the VHVs.

Fourthly, the study found that the performance of VHVs was similar to other health care provider which is an encouraging sign for the program because they are the key grassroot-level players in CCM of malaria. This is also very important in view of the fact that they work in the hard-to-reach areas despite several challenges as outlined in this paper. A recent external evaluation of NMCP by WHO Myanmar has also appreciated the role of VHVs in providing access to malaria diagnosis and treatment in the far-flung areas [16]. Another countrywide analysis of the performance of VHVs showed that malaria care provided by VHV was as good as BHS [26].

The study had the following strengths. Firstly, this was a national-level assessment of VHV performance using routine program data which makes it generalizable, replicable to other settings, and reflective of the true situation. Secondly, a mixed methods approach not only brings out the gaps in the performance of VHVs but also enables us to understand the challenges faced by the volunteers and solutions to mitigate them. Thirdly, the study also adhered to “STrengthening the Reporting of OBservational Studies in Epidemiology” and COREQ guidelines to report the study findings [27, 28].

There were a few limitations as well. Firstly, the study did not assess the role of VHVs under other implementing partners (i.e., those not under the NMCP). The qualitative study did not cover the large ethnic and linguistic variations in the communities raising issues of generalizability, thus warranting future qualitative studies. Secondly, completion of treatment and adequate dosing of anti-malarial drugs was not studied here due to the lack of reliable data. Thirdly, health care workers may have been reluctant to critically comment on the shortcomings of the program as it might have an impact on their jobs. Fourthly, secondary data from routine program records was used in this study, and there was no means of validating the data.

Several work-related and logistic challenges have been listed in this paper. In view of these, specific recommendations have been proposed for the program. Firstly, although there was a good community acceptance for the VHVs, poor community support was recognized as a key barrier in some communities due to the contextual factors such as language, religion, and lack of interest of the village leader in health-related activities which could be tackled locally by context-specific recommendations. Some health staff has recommended that VHVs should be given some general medicines like paracetamol, oral rehydration salt, and multivitamins to build trust through the provision of basic clinical services in the community. A welcome step has been made in this regard recently where the volunteers are now been given more roles in other diseases such as tuberculosis, HIV, leprosy, dengue, and lymphatic filariasis as an integrated community malaria volunteer. Village leaders and community should be advocated about the volunteers’ work by arranging a village meeting or during the health activities done by the BHS to ensure community support as said by few health care providers.

Secondly, transportation difficulties and the costs associated with it have been cited as a key challenge to timely reporting, referral, and case management of malaria by the VHVs working in difficult terrains as reported by another study in Myanmar [29]. Thus, it was also suggested by the VBDC staff to give more transportation allowance to the VHVs and also recruit more VHVs in such areas. Finally, the distribution of VHVs could be reconsidered taking into account the burden of malaria, geographical reach, and other local aspects in different states/regions.

## Conclusion

VHVs play an important role in malaria diagnosis and management in Myanmar, especially in the hard-to-reach areas, with their performance comparable to other health care providers. More programmatic support to VHVs is needed in terms of logistics, transportation allowance, and supervision to improve CCM of malaria in remote areas.

## Abbreviations

ACT: Artemisinin combination therapy
BHS: Basic health staff
CCM: Community case management
FGD: Focus group discussion
GMS: Greater Mekong Subregion
KII: Key informant interview
NMCP: National Malaria Control Programme
RDT: Rapid diagnostic test
VBDC: Vector-borne disease control
VHV: Village health volunteer
