# Correction to: How are the village health volunteers delivering malaria testing and treatment services and what are the challenges they are facing? A mixed methods study in Myanmar

**DOI:** 10.1186/s41182-018-0120-y

**Published:** 2018-11-07

**Authors:** Nay Yi Yi Linn, Jaya Prasad Tripathy, Thae Maung Maung, Khine Khine Saw, Lei Yee Win Maw, Badri Thapa, Zaw Lin, Aung Thi

**Affiliations:** 1 Vector Borne Disease Control Program, Ministry of Health and Sports, Nay Pyi Taw, Myanmar; 2 International Union Against Tuberculosis and Lung Disease, The Union South East Asia Office, New Delhi, India; 3 International Union Against Tuberculosis and Lung Disease, Paris, France; 4 Department of Medical Research, Ministry of Health and Sports, Yangon, Myanmar; 5 Independent Public Health Consultant, Yangon, Myanmar; 6 Malaria Unit, World Health Organization Country Office, Yangon, Myanmar

## Correction

In the original publication of this article [1], the article title should be changed to “How are the village health volunteers delivering malaria testing and treatment services and what are the challenges they are facing? A mixed methods study in Myanmar”.
